# Acoustic myography of the biceps and triceps brachii muscles during retrieving drills in field trial dogs

**DOI:** 10.3389/fvets.2026.1859593

**Published:** 2026-07-06

**Authors:** Anna Kristina Hugel, Jane Manfredi, Julia Tomlinson

**Affiliations:** 1 Twin Cities Animal Rehabilitation + Sports Medicine, Burnsville, MN, United States; 2 College of Veterinary Medicine, Department of Large Animal Clinical Sciences, Michigan State University, East Lansing, MI, United States

**Keywords:** AMG, biceps tendinopathy, canine, lameness, muscle function, speed

## Abstract

**Introduction:**

Field-trial dogs decelerate from speed, turn, and retrieve birds, potentially predisposing to biceps tendinopathy. Acoustic myography (AMG) measures muscle contraction. We hypothesized that braking/turning (turn) to retrieve birds produces more biceps and triceps work vs. gallop to the bird (out-run), or back with the bird (return) and that work in these muscles would be asymmetric between limbs at turn.

**Methods:**

Fourteen conditioned, orthopedically sound retrievers were fitted with wired sensors adhered bilaterally to triceps and biceps, connected to a harness holding AMG equipment. Dogs performed three 91.44 m retrieves with 2 s AMG recordings sampled mid out-run and return, and 1 s during turn; speed was calculated for each section. Data analysis: Shapiro-Wilk tests for normality and either Student's *t*-test (AMG data inside vs. outside leg at turn) or one-way mixed effects model with Geisser-Greenhouse correction or ANOVA with Tukey's multiple comparisons *post-hoc* test (other AMG parameters, dog speed). Significance was p < 0.05.

**Results:**

Speed was faster at out-run vs. return. Biceps at out-run had greater amplitude vs. return and frequency vs. turn. Biceps at turn had greater amplitude than return. Triceps at both out-run and turn had greater amplitude vs. return. Contraction frequency was greater for biceps at out-run vs. turn and for triceps at turn vs. out-run.

**Discussion:**

Carrying a bird is not a risk factor for biceps tendinopathy in field trial dogs; excess tensile load on the biceps from triceps action at turn, and high muscle work in the biceps at out-run are likely risk factors.

## 1 Introduction

Thoracic limb lameness is common in active dogs that perform repetitive tasks (1–3), and biceps tendinopathies are reported as a common cause of thoracic limb lameness (4–10). In one standalone sports medicine and rehabilitation veterinary specialty clinic, hunting retrievers made up 25% of the clinic population but 43% of the cases of biceps tendinopathy (4). Hunting dogs are required to hold a weight in their mouth, whether a bird or a dummy. Carrying a mouth weight has been shown to shift weight bearing to the thoracic limbs (4, 11) and may predispose these dogs to muscle fatigue in their thoracic limbs (11, 12). In fact, carrying a mouth weight resulted in an increase in muscle work in the biceps brachii relative to the brachiocephalicus the walk in hunting retrievers (4). Muscle fatigue with chronic overuse has been shown to predispose horses to tendinopathy, and this may occur in dogs as well (12). Canine thoracic limbs principally have a braking function (13, 14). The biceps and triceps have opposing actions at the elbow though both work to stabilize the elbow joint during stance (15). The biceps brachii also stabilizes the shoulder joint when weight bearing (15). Hunting retrievers typically move at a gallop when retrieving; electromyographic studies on biceps and triceps of dogs at the gallop show the biceps is active approximately 23% of total stride; the last 44% of stance and first 16% of swing (15). The triceps lateral head, in comparison, is active at foot strike and the first 70% of stance (20% of total stride) (15). These two muscles are therefore co-contracting for 14% of stance phase (15). While there are electromyographic (EMG) studies of action of these muscles at the gallop (15), there is no study of the action of these muscles when braking and turning. As the forelimbs have braking function, it is possible that the act of braking and turning from a gallop requires more biceps and/or triceps muscle work than that of a gallop alone (with or without carrying a bird). It is certainly true for the forelimb retractor latissimus dorsi during braking in a study of dogs running on a treadmill (14) and co-contraction of the biceps and triceps would be needed to fix the elbow during braking. This additional muscle work could directly cause fatigue in the musculotendinous unit of the biceps thus predisposing to tendinopathy (12). An alternative possibility is that increased triceps work during turning places increased tensile load on the biceps due to the oppositional actions of these muscles at the elbow. The biceps tendon is already under tensile load in the stance phase, regardless of muscle action, as the act of shoulder flexion applies tension to the biceps tendon (16) thus, the biceps tendon limits translation of the shoulder joint in flexion, providing stability (17). The triceps being a much larger muscle than the biceps likely creates more force when contracting, but no direct studies on force production of the biceps and triceps are available in the dog.

Acoustic myography (AMG) has been validated (18) and performed in research on both healthy and diseased dogs (4, 18–25). Acoustic myography measures the direct sounds of muscle contraction, correlating directly to force of contraction in both isometric and dynamic muscle contraction (26, 27). When muscles contract, they create tiny vibrations; active muscles make pressure waves that reach the skin above the muscle, where they can be measured. The AMG signal is noise-free during periods of inactivity. As with electromyography (EMG), records the muscle parameters of temporal and spatial summation as well as on:off ratio (4, 18–21). Both temporal and spatial summation, as measured via AMG, have been shown to directly correlate with force production (28). A key difference between AMG and EMG is that EMG measures action potentials for nerves, rather than direct muscle parameters, and does not correlate to force during movement (27–30); acoustic myography measures the sound of muscle contractions (25). These sounds provide the basis for E (efficiency or on:off ratio), S (spatial), and T (temporal) scores. During a specific task, a high E-score (scale 1–10) represents a low on:off ratio (21). A high S-score (scale 1–10) represents low signal amplitude or less fibers recruited during work (21). A high T-score on a scale of 1–10 represents low frequency of contraction (21).

The aim of this study was to evaluate the muscle function of the biceps and triceps brachii while field trial dogs are performing retrieving drills as a potential explanation for the occurrence of biceps tendinopathy in this group of dogs. We hypothesized that braking and turning to retrieve a bird would result in greater muscle work of the biceps and triceps brachii as compared to gallop with or without a bird in the mouth. Additionally, we hypothesized that the work in these muscles would be asymmetric between limbs at turn.

## 2 Materials and methods

### 2.1 Selection criteria

The inclusion criteria toparticipate in the study were as follows: a retriever breed dog, between 2 and 8 years of age, between 50 and 75 lbs. (22.73–34.09 kg), and free of detectable soft tissue or orthopedic injuries. Dogs also needed to be able to complete the trial sessions while staying on course. The fourteen dogs included were client owned and handled by their owner (termed amateur; *n* = 6 dogs, 6 handlers) or handled by a professional handler who housed them for training (termed professional; *n* = 8 dogs, 1 handler). Clinical orthopedic disease of the limbs was ruled out via orthopedic examination and gait analysis on a pressure sensitive walkway. ^1^

### 2.2 Orthopedic evaluation and gait analysis for inclusion in study

All dogs underwent orthopedic examination performed by a Diplomate of the American College of Veterinary Sports Medicine and Rehabilitation (JET). The dog's brachial and thigh circumferences were measured using a spring weighted tape measure^2^ performed three times per limb with the average measurement used (31). Each dog underwent goniometry of the thoracic and pelvic limb joints to evaluate passive range of motion. Biceps brachii stretch, measured as the maximal degree of elbow extension with the shoulder in full flexion, and shoulder joint passive abduction in end range extension, were measured via goniometry. If no abnormalities [difference of 10 degrees or greater for goniometry or circumference difference greater than 0.5 cm (32, 33)] were detected, each dog underwent gait analysis using a pressure sensitive walkway to evaluate for lameness. The pressure sensitive walkway was previously validated and calibrated by the manufacturer (34, 35). Each dog was acclimated to the walkway for a 5-min period prior to any measurements. Professional dogs were handled by the same handler (Isaac Langerud, IL) and amateur dogs were handled by their respective owner-handler for the study. Dogs were trotted on the pressure sensitive walkway several times to obtain three valid passes at a trot. A valid pass was recorded if the dog did not step off the mat, trotted in a straight line, and had three gait cycles recorded for each pass with a consistent gait (<10% variability in velocity in a single pass). A ≤ 6% difference in Total Pressure Index (TPI) was accepted as normal between each forelimb and each hindlimb during evaluation (36–38). Only dogs that were deemed orthopedically normal based on the subjective and objective examination above were allowed to be enrolled in the study. Comparison of the gait parameters of TPI and step length was performed with and without the harness and equipment to rule out any effect of the equipment (harness, adhesive tape, AMG sensor, AMG recording device, and gel) on step length or TPI prior to AMG data collection.

### 2.3 Field retrieve environment

**Figure 1:**
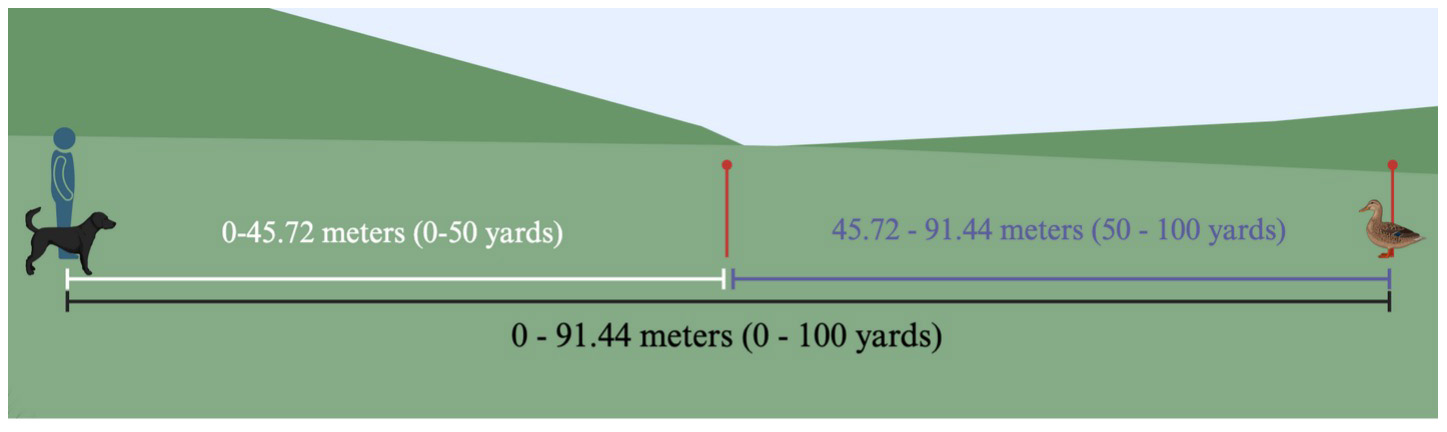
‘Graphical representation of one of the retrieving drills with distances marked. Physical markers were placed to indicate the start, halfway, and retrieve.” created in BioRender. Hugel, K. https://BioRender.com/invk7af is licensed under CC BY 4.0.

Retrieves were performed over a 91.44-meter (100 yard) distance on flat grassland; either on wet 5-inch grass (amateur), or dry 2-inch grass (professional facility). The track had markers placed at the 0, 45.72 (50-yard), and the 91.44-meter (100-yard) level (Figure 1). Dogs were placed in a heel position with their handler at the 0-meter marker. The AMG recording device was turned on by the handler and a dead bird weighing between 1.36–2.27 kg (3–5 lbs.) was dropped from a high hold (visible to dog) to land within 1 foot of the 91.44-meter marker, just prior to the dog being released to perform the retrieve. The 91.44-meter run to the bird was termed *out-run*, the duration of braking, turn and bird prehension was termed *turn*, and the run back to the 0-meter mark was termed *return*. Runs that deviated more than 0.61 meters (2 feet) from the straight out-run or return path, or when the dog did not retrieve within 0.91 meters (3 feet) of the 91.44-meter mark (over-ran) were excluded from the study.

### 2.4 Speed data

The handler remained standing at the 0-meter marker. Individual stopwatches were used to record A) total run time, B) time between 0 and the half way (45.72-meter) marker, C) time between 45.72 and 91.44 meter marker, D) time for total out-run (0 to 91.44-meter marker), E) time (post-retrieval) returning between the 91.44 and 45.72 meters, F) time between 45.72 and 0-meters, and G) the time for total return run (91.44 to 0-meter marker). Physical markers were used to guide stopwatches. Recorded times were used to accurately sample AMG data, in addition to the speed calculation for each running segment (distance in meters/time in seconds). The time taken for deceleration, prehension of bird and turn was calculated by subtracting out-run (0 to 91.44 meter) time plus return (91.44 to 0 meter) time from total run time.

### 2.5 Acclimation and placement of AMG sensors

**Figure 2:**
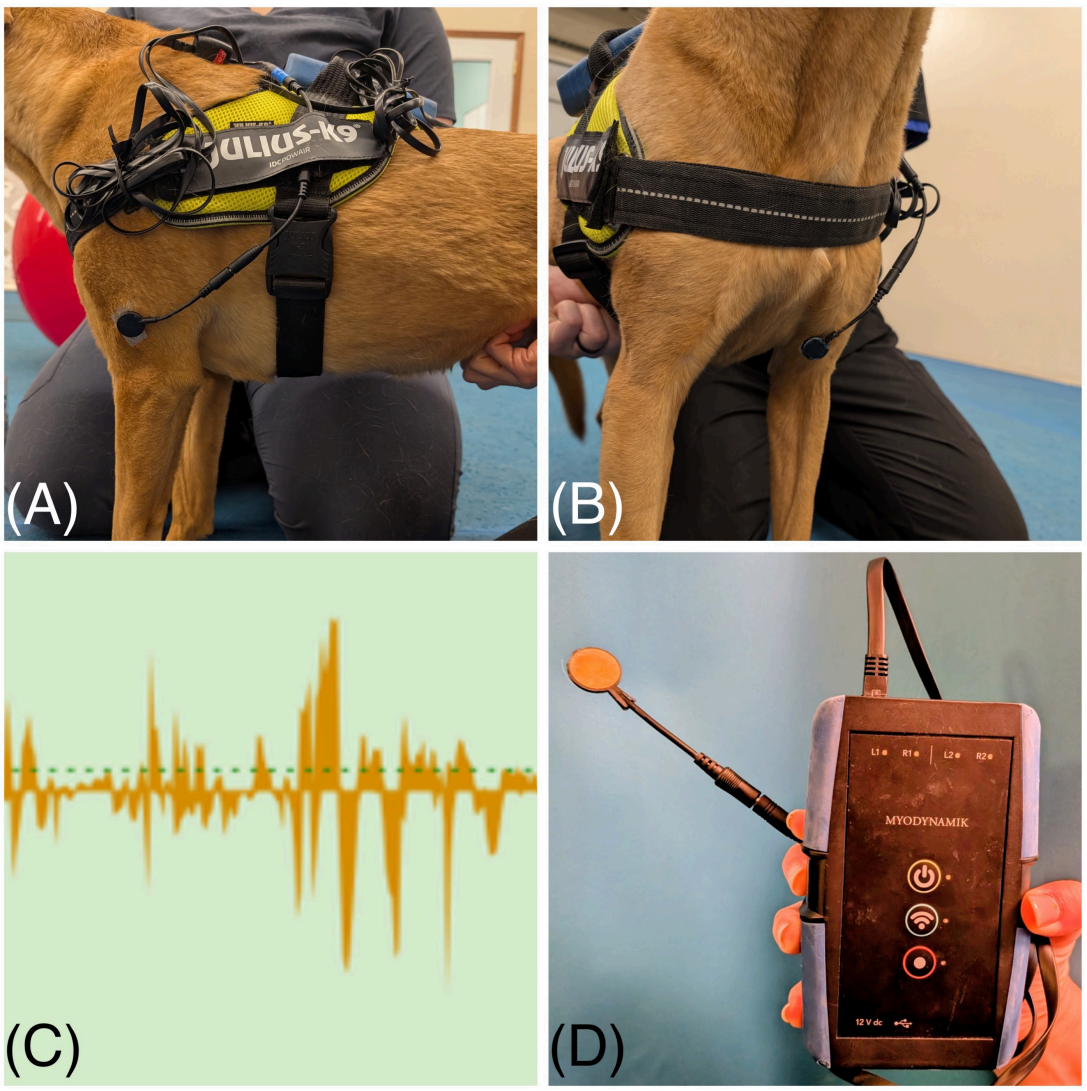
Demonstration of AMG sensor placement with harness including recording unit and wires **(A)** at 1/2 humeral length for triceps brachii and **(B)** at 2/3 humeral length for biceps brachii (sensors are adhered in A and B with sticky-backed tape for demonstrations only). This is an example dog and was not included in the study. **(C)** Sample of an AMG recording. **(D)** Picture of AMG device with 20 mm sensor.

Dogs wereacclimated to a harness^3^ for 3 training days (routine retrieves) prior to data recording while wearing a dummy weight (1 lb. block) that exactly simulated the AMG recording device size and weight. For the real data trials, the actual AMG recording device was fixed to the harness under the handle (Figure 2).

Areas of approximately 2.5 square cm (1 square inch) were clipped using a #40 blade at the location where the sensors were to be placed. A small amount of acoustic coupling gel^4^ was placed on the skin and on the sensor. Wired AMG sensors^5^ were placed on the biceps brachii (at 2/3 humeral length) and the lateral head of the triceps brachii (at ½ humeral length) bilaterally, adhered using scrim backed, pressure-sensitive tape^6^, and connected to the AMG recording device affixed under the harness (Figure 2).

### 2.6 Acquisition of data

The AMG signal was transmitted via an internal WiFi signal to a handheld computer tablet^7^. AMG recording parameters were set at a sampling rate of 1,000 Hz, 10-bit resolution, and thresholds set to previously published parameters (4). The threshold was adjusted upwards from 0 to a maximum value of 0.28 until the AMG scores approached a steady state, ideally without any of the scores reaching 0 or 10 (maximum value). Additionally, maximum frequency (max T) was set to 160 Hz (20).

Five runs were performed, and the data from two to three of the most consistent retrieves (no dropping of the bird, no excess time to find the bird) were used for analysis provided the duration of each segment (i, ii, and iii, outlined below) for an individual was within two standard deviations of the mean. During each retrieving drill, direction of turn was noted to determine the inside and outside thoracic limb. A 5-min rest period occurred between drills to prevent fatigue and any cumulative effects on muscle activity (19, 39). This is consistent with training methods used by all nine handlers. Samples of myographic recordings for each of the valid retrieves were analyzed from:

- 2 s at mid out-run (including the time the dog was at the 45.72-meter marker)
- Turn (braking, turning, and bird prehension) with data separated to inside and outside leg of turn. Sampling duration was chosen based on mean turn time for all dogs
- 2 s at mid return (including the time the dog was at the 45.72-meter marker)

### 2.7 Statistical analysis

Prior to thestudy, sample size to see a significant difference in the AMG data within a muscle between out-run, turn, and return was computed with an alpha of 0.05, 80% power and a large effect size (Cohen's *f* = 0.4) was determined to be 12 dogs. We included additional dogs to account for any issues with the dogs or data collection. Data were assessed for normality using a Shapiro-Wilk test and then analyzed with either a one-way mixed effects model with Geisser-Greenhouse correction or an ANOVA with Tukey's multiple comparisons *post-hoc* test (speed and all AMG data apart from turn inside vs. outside leg) and a Student's t-test (turn inside vs. outside leg AMG data). Significance was set at *p* < 0.05.

## 3 Results

### 3.1 Included dogs

A total of 14 dogs met the inclusion criteria for the study. The subjects were 2 Chesapeake Bay Retrievers and 12 Labrador Retrievers. Mean body weight was 27.5 kg ± 2.56 kg (60.5 ± 5.64 lbs.) with a mean body condition score of 4.5 ± 0.5 out of a 9-point system^8^ and the mean age was 4.5 ± 2.3 years old. There were 7 female dogs and 7 male dogs.

### 3.2 Gait analysis (no effect of harness)

There was no significant effect of harness and equipment on the gait parameters of TPI (*p* = 1.0) and step length (*p* = 1.0). In each case the data was normally distributed.

### 3.3 Speed

**Figure 3:**
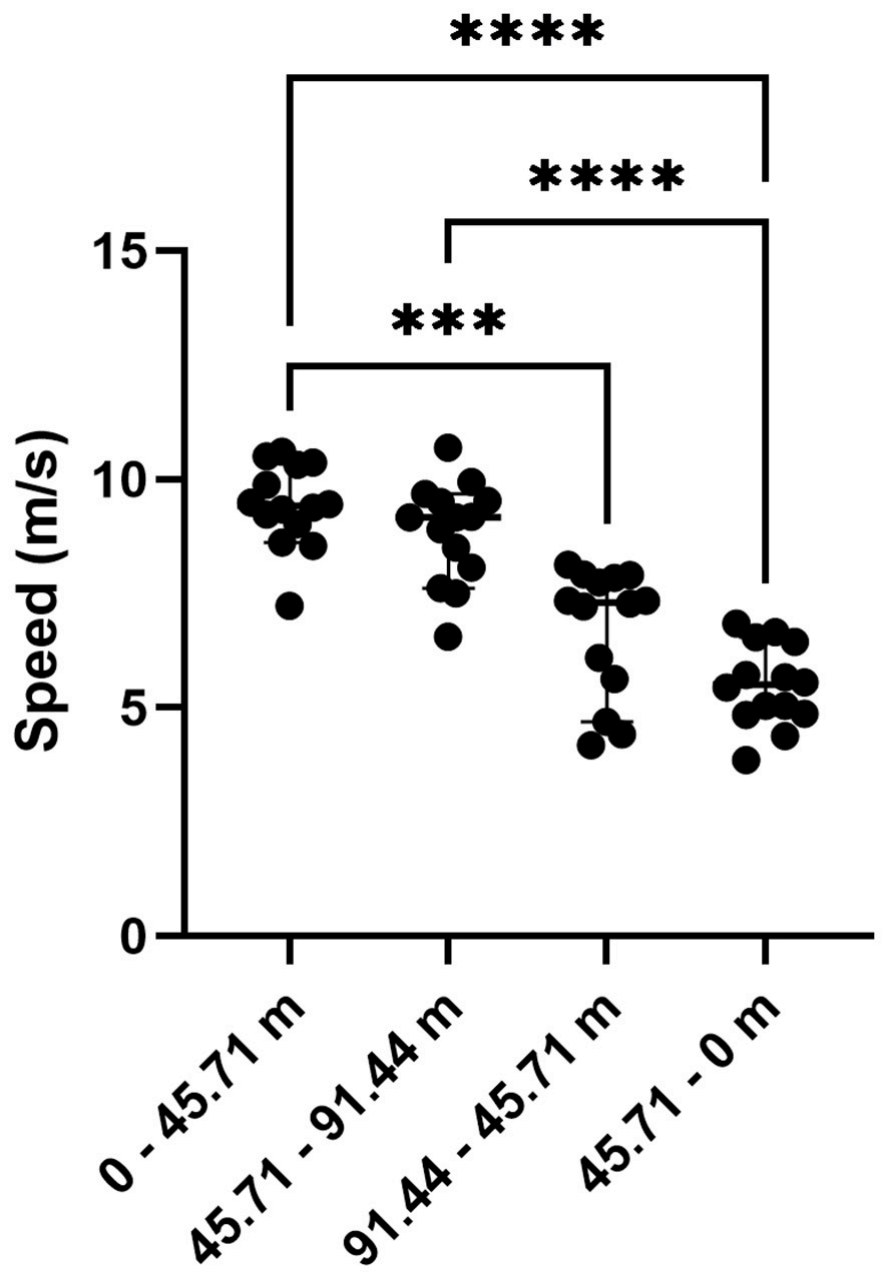
Calculated speed (m/s) during each 45.71 meters of the trial either before the retrieve (0–45.71 m and 47.51–91.44 m) or after (91.44–45.71 and 45.71–0 m) (*n* = 14). ^****^p = 0.0001, ^***^p=0.0008.

Mean speed of the out-run was 9.0 ± 1.0 m/s. Mean speed of the return was 5.9 ± 1.1 m/s, with sections of the out-run being significantly faster than sections of the return (all *p* ≤ 0.001) (Figure 3).

### 3.4 Turn details

Deceleration was visualized after the dogs passed the 91.44-meter marker when the bird was identified by the dog. Mean duration of turn was 0.71 ± 0.38 s, AMG sample length for turn was 1 second. Four dogs turned consistently left, 4 dogs turned consistently right, and 6 dogs turned either direction depending on the run (4/4 amateurs; 2/6 professionals).

### 3.5 AMG data

There was no significant difference between inside and outside leg muscle parameters at turn, subsequently the data was analyzed with the inside and outside legs averaged together to compare to out-run and return (Tables 1, 2).

**Table 1:** Mean AMG values with standard deviation for out-run, turn (separated into outside and inside legs), and return for biceps and triceps brachii.

Drill segment	Muscle sampled	E mean ±SD	S mean ±SD	T mean ±SD
Out-run	BB	3.05 ± 2.22	5.40 ± 1.95	7.71 ± 0.57
	TB	2.34 ± 1.12	5.74 ± 1.66	7.80 ± 0.67
Turn	BB outside leg	4.57 ±2.38	5.10 ± 1.88	7.05 ± 1.33
	TB outside leg	4.74 ± 1.53	6.01 ± 2.93	7.19 ± 1.79
	BB inside leg	4.01 ± 2.17	4.50 ± 2.66	6.53 ± 1.83
	TB inside leg	3.51 ± 2.16	3.72 ± 2.08	6.82 ± 1.47
Return	BB	3.46 ± 1.70	6.66 ± 1.82	7.72 ± 1.17
	TB	3.17 ± 1.15	6.93 ± 1.33	7.40 ± 0.83

**Table 2:** Mean AMG values with standard deviation for out-run, turn, and return for biceps and triceps brachii.

Drill segment	Muscle sampled	E mean ±SD	S mean ±SD	T mean ±SD
Out-run	BB	3.05 ± 2.22	5.40 ± 1.95	7.71 ± 0.57
	TB	2.34 ± 1.12	5.74 ± 1.66	7.80 ± 0.67
Turn	BB	4.14 ± 1.49	4.88 ± 2.11	6.71 ±1.43
	TB	3.96 ± 1.31	4.64 ±1.88	6.98 ±1.10
Return	BB	3.46 ± 1.70	6.66 ± 1.82	7.72 ± 1.17
	TB	3.17 ± 1.15	6.93 ± 1.33	7.40 ± 0.83

#### 3.5.1 Biceps brachii

**Figure 4:**
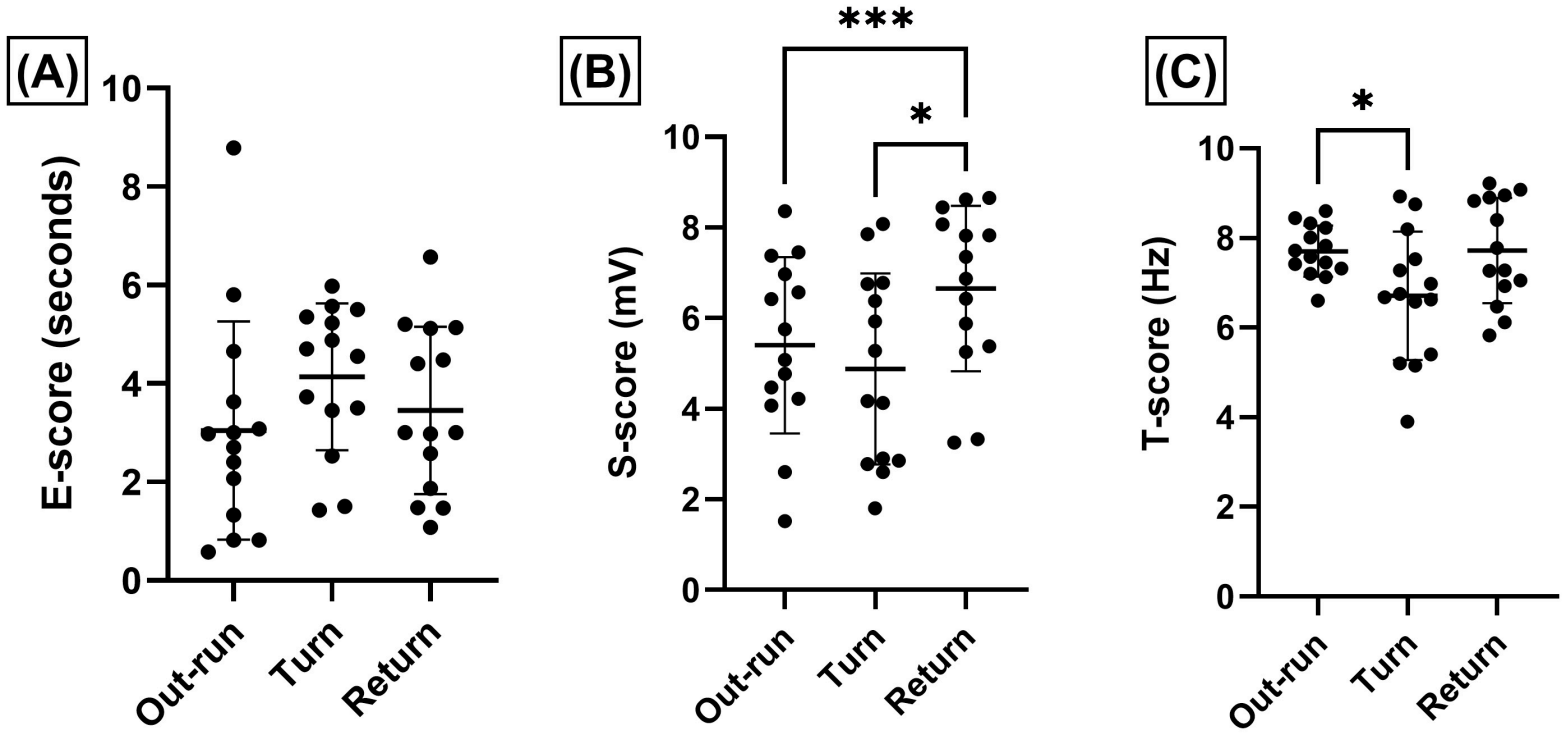
**(A)** E-score (seconds), **(B)** S-score (mV), and **(C)** T-score (HZ) for biceps brachii (BB) muscle at out-run, turn, and return (*n* = 14). ^***^p = 0.0008, ^*^p = 0.017 and *p* = 0.03.

The S-score of the biceps of the out-run was significantly lower than that of return (p < 0.001) (Figure 4), indicating greater number of fibers recruited for the out-run. The S-score of the turn was significantly lower than the return (*p* = 0.017) (Figure 4), indicating greater number of fibers recruited at turn than when returning with the bird. The T-score of the out-run was significantly lower than the turn (*p* = 0.03) (Figure 4), indicating greater frequency of contraction during out-run. No other comparisons were significant for E, S, or T scores (all *p* > 0.05) (Figure 4).

#### 3.5.2 Triceps brachii (lateral head)

**Figure 5:**
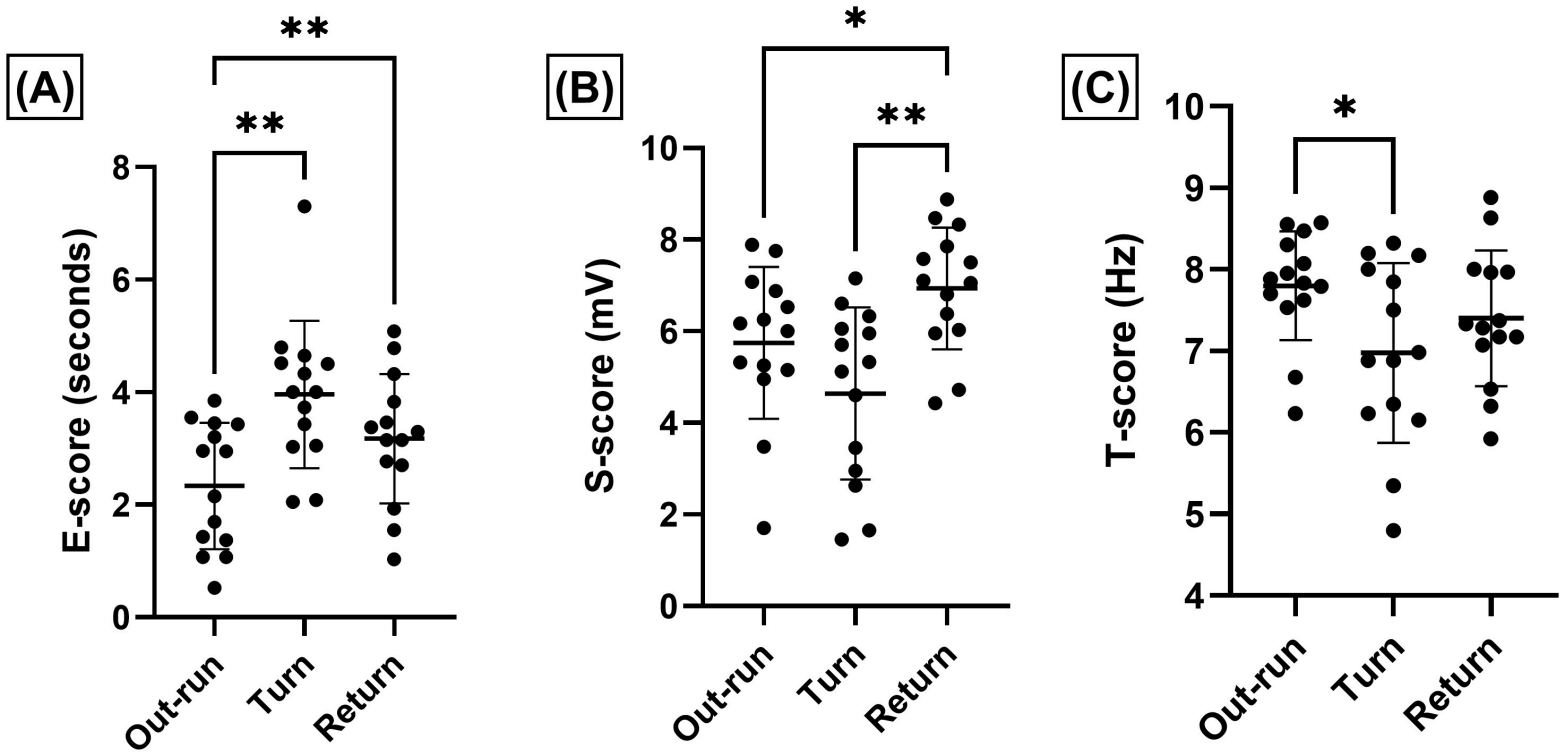
**(A)** E-score (seconds), **(B)** S-score (mV), and **(C)** T-score (HZ) for Triceps Brachii (TB) muscle at out-run, turn, and return (*n* = 14). ^**^p = 0.005, *p* = 0.003, and *p* = 0.002, ^*^p = 0.04 or 0.047.

Both the E- and S-scores of the triceps at out-run were significantly lower than the return (*p* = 0.005 and *p* = 0.04, respectively) (Figure 5), indicating greater duration of contraction and larger numbers of fibers recruited during out-run. The E-score at out-run was significantly lower than turn (*p* = 0.003) (Figure 5), indicating greater duration of contraction during out-run. The S-score at turn was significantly lower than return, indicating larger number of fibers recruited (*p* = 0.002) (Figure 5). The T-score at turn was significantly lower than out-run, indicating higher frequency of contraction at turn (*p* = 0.047) (Figure 5). Other comparisons were not significant (all *p* > 0.05) (Figure 5).

## 4 Discussion

We partially reject our hypothesis that the act of braking and turning to retrieve a bird produces more work in the biceps and triceps muscles compared to gallop with or without a bird, as it was accepted only in the case of the triceps at turn. We fully reject our hypothesis that work between the inside and outside leg was asymmetric for the studied muscles.

### 4.1 AMG findings

The greater spatial summation found in both muscles at out-run and turn as compared to return indicates these tasks recruit a larger amount of muscle fibers than when carrying a bird, and that the latter is not the most difficult of the three tasks studied. This leaves us with the likely risk factors for biceps injury in field dogs (if due to muscle work) being fast sprinting, turning, or a combination of the two.

In the case of the biceps, there was significantly greater frequency of contraction at out-run vs. turn. Both spatial and temporal summation are directly related to force of contraction in AMG studies in humans (28), correlated with force production in toad muscle (40), and acoustic myography signals show asymmetry between hindlimbs in a lame horse (low contraction amplitude in affected limb) which correlated to reduced rate of acceleration in that limb (41). We presume it will also correlate to force production in dogs. AMG frequency is also known to be higher at faster speeds (28), therefore the finding of higher frequency at gallop in the biceps when compared to deceleration and turn could be explained solely by speed, however we did not get similar findings in the triceps. Triceps frequency of contraction was actually greater at turn, indicating more muscle work than at out-run. Given the triceps is a much larger muscle than the biceps and is its antagonist, it presumably produces greater force (42, 43) which could put additional strain on the biceps tendon during the action of turning. Increased triceps muscle work was also found during a skijoring task as compared to free running in our prior study (44) and was postulated to be a possible cause of excess tension on the musculotendinous unit of the biceps due to its opposing action.

The task of running fast out to the bird could be the risk factor for biceps tendinopathy in retrieving dogs given the greater spatial summation at out-run vs. return, and greater temporal summation at out-run vs. turn. If speed were indeed a risk factor for biceps tendinopathies, one would expect other canine speed sports to have a high incidence of this disorder. Despite a 69.9% soft tissue injury rate in racing Greyhounds, mostly muscular (not tendon or ligament) injury was reported with a short rest period before successful return to racing (45). Greyhounds run at 17 m/s (46), almost double what the dogs in the current study ran during out-run (9 m/s). In the authors' sports medicine and rehabilitation specialty practice, sporting Whippets comprised 1% of the clinic population, but only 0.05% of the cases treated for biceps tendinopathy over 19 years. Whippet patients that were treated for biceps tendinopathy participated in flyball (a sport with 180-degree turns) and not in other speed sports, such as coursing, which makes speed alone an unlikely risk factor for clinically presenting biceps tendinopathy. The speed at which the retrieving dogs in the present study ran on the out-run was also slower than both skijor dogs (15 m/s at free run) from a previous study by the authors (44) as well as reports from racing Greyhounds (46).

Duration of contraction was higher in the triceps at out-run vs. both turn and return; longer duration of contraction indicates more re-recruitment of fibers but has not been directly linked to force production in acoustic myography.

The difference in muscle work (spatial summation) in both the biceps and triceps between out-run and return could be solely explained by the slower speed during return with the bird. In the authors' previous AMG study on skijoring dogs (44), biceps brachii work during free-run was not significantly higher than during the task of skijoring, despite the free-run being 1.3 times faster than the skijor task (authors speculated that the tasks may have been equal muscle work but resulting in a slower speed during skijor). Based on these prior AMG findings, the authors of the current study expected, and did find a similarly slower pace during return carrying the bird, but we did not find equal muscle work between these two tasks. It is not clear whether the dogs slowed during return due to having achieved their reward (the bird) or whether the weight of the bird necessitated slowing speed to conserve energy. The slower speed of return could be due to energy conservation secondary to fatigue, which is unlikely given these dogs are trained to run much further (up to 300 yards or 274.32 meters). Conversely, it is likely that the dogs had the muscle capacity to run faster with the bird but chose not to for other reasons such as reduced drive. Regardless of the cause of the slower return run, the experimental setup mimicked real life training and trialing for field dogs, and it was noted by handlers that a slower return run was a normal expectation.

### 4.2 Turn dynamics

The majority of dogs were consistent in the direction of turn which is likely representative of the general hunting retriever population. However, in this study there was no significant difference between inner and outer limb muscle action. The lack of difference in muscle action between inner and outer leg biceps and triceps was surprising, as two studies of turning in dogs (47, 48) showed a difference between outer and inner limb function; with the inner limb having more shock absorbing and braking function (more compression via elbow flexion with carpal extension, and longer stance). The outer limb having a large overall ground reaction force and being more propulsive (stiffer and more shoulder extension). It is likely that these asymmetric forces during turn (47) are mediated by muscles other than those examined in the present study, though no myographic studies of such a turn have been published.

If asymmetric action during turning involving muscles not examined in this study was primarily responsible for overload and subsequent biceps pathology, one would expect a clinical presentation of unilateral lameness and pathology rather than bilateral. The lack of discrepancy in muscle action between inside and outside legs found in this study is supportive of reported clinical findings of bilateral biceps tendinopathies (6, 49). A recent retrospective study evaluating a treatment of biceps tendinopathies diagnosed by musculoskeletal ultrasound in 30 dogs found that in most cases (27 dogs) the tendinopathy was bilateral (6). That study evaluated dogs from all disciplines, with 70% of cases being either hunting or agility dogs; both sports that involve turning (6).

## 5 Limitations

Limitations include that dog speed was not possible to control, and the differences in muscle contraction parameters of temporal and spatial summation could in part be due to speed of motion. Professional dogs were far more consistent in turn direction and time, and if we had used only professional dogs, we may have had different results, however the number of professional dogs available to us was limited. The differences in grass height from long with moisture vs. short without moisture could have affected traction and therefore could have prolonged turn time for the amateur dogs who were on the wetter surface.

## 6 Conclusion

Carrying a bird when retrieving is not a risk factor for biceps tendinopathy in field trial dogs. Excess tensile load on the biceps from triceps action at turn, and high muscle work in the biceps at out-run are likely risk factors for the disorder. Future research should include a kinematic study of dogs retrieving a bird coupled with myography.

## Data Availability

The raw data supporting the conclusions of this article will be made available by the authors, without undue reservation.

## Footnotes

1 Gait4dog, CIR systems Inc, Franklin, NJ, USA

2 Gulick II, Warrenville, IL, USA

3 Julius-K9 IDC, Powerharness

4 Ekkomarine Medico A/S, Holstebro, Denmark

5 20 mm diameter; MyoDynamik sensors, Copenhagen, Denmark

6 3M, Maplewood, MN

7 iPad, Apple Inc., Cupertino, CA

8 Nestlè PURINA Body Condition System
